# Racial differences in food insecurity among South African adults in 2021: a decomposition analysis

**DOI:** 10.11604/pamj.2026.54.33.47182

**Published:** 2026-06-08

**Authors:** Lungile Gift Gretel Nkosi, Olalekan Ayo-Yusuf

**Affiliations:** 1 Africa Centre for Tobacco Industry Monitoring and Policy Research, School of Health Systems and Public Health, University of Pretoria, Pretoria, South Africa

**Keywords:** COVID-19, food insecurity, South Africa, racial differences

## Abstract

**Introduction:**

the COVID-19 pandemic worsened global food insecurity and deepened socio-economic gaps, particularly in South Africa. This study aimed to examine the socio-economic factors contributing to racial differences in self-reported food insecurity and examine the intersectionality of race and socioeconomic status in the context of food insecurity in South Africa.

**Methods:**

we analysed data from the 2021 South African Social Attitudes Survey (n = 2,837), a nationally representative survey that employs multistage probability sampling to capture the adult population aged 16 years or older. Food insecurity was assessed using a validated one-item measure. Statistical analyses included Blinder-Oaxaca decomposition and Poisson regression, with statistical significance set at p < 0.05.

**Results:**

the prevalence of food insecurity was 39.5% (95% CI = 36.0-43.1). Compared to those who self-identified as White, those who self-identified as Black Africans were more likely to report food insecurity (aPR = 1.88; 95%CI = 1.32 - 2.69). Among employed individuals, food insecurity was higher among Black Africans (32.7%) than other race groups (19.8%). Racial disparity in food insecurity widened among the unemployed (48.9% among Black Africans vs. 28.5% among others). Decomposition analysis suggests that employment accounted for 25% of the disparity that remained an 'unexplained' (discriminatory) significant contributor to racial differences in food insecurity in favour of other 'race groups'.

**Conclusion:**

the study underscores the effect of multiple marginalisation and in particular, the complex interplay between socio-economic factors and racial disparities, in driving food insecurity in South Africa, emphasizing the need for targeted policy interventions.

## Introduction

The COVID-19 pandemic had far-reaching consequences for global food security and public health, prompting concerns about the exacerbation of existing socioeconomic disparities. The number of people experiencing moderate or severe food insecurity rose from approximately two billion in 2019 to 2.4 billion in 2020, representing about one in three people worldwide [1]. This increase reflects a significant shift, particularly in Europe, which reported a surge in food insecurity, marking a notable departure from the declining trend observed since 2014 [1]. These alarming trends are attributed to the stringent lockdown measures implemented worldwide to contain the spread of the virus. While these measures were necessary from a public health perspective, they led to widespread closures of businesses and unprecedented job losses, resulting in a substantial reduction in household incomes across the globe [2-5].

In South Africa, the economic fallout of the pandemic was particularly severe [6]. Official data revealed a staggering increase in unemployment, with approximately 2.24 million jobs lost between March and June 2020 [6]. In response, the government introduced the Social Relief of Distress (SRD) grant in May 2020 to support those who were unemployed or had lost their jobs due to COVID-19 [7,8]. During 2021, SRD grant recipients reported spending it primarily on essential items, with food accounting for 93.3% of respondents' expenditures, followed by electricity (31.9%) and face masks (17.3%) [7]. Meanwhile, the cost of essential food items surged, with the household food basket reportedly increasing by 7.8% in the first three weeks of the lockdown [9].

These economic shocks disproportionately affected vulnerable populations, as shown by epidemiological data indicating that one in five South Africans experienced food insecurity in 2021 [10]. The impact was most severe among those who self-identified as Black Africans and those who self-identified as Coloured, with prevalence rates of 22.6% and 24.2%, respectively [10]. This aligns with current levels of poverty defined as living below the upper-bound poverty line of R992 (~$55) per capita per month across different race groups [9]. Black Africans have been reported to have the highest level of poverty (46.6%) compared to those who self-identified as Coloured (32.3%), Indian/Asians (4.6%) and White individuals (0.8%) [11]. Similarly, unemployment levels during 2023 were highest among Black Africans (36%), followed by Coloured (22%), Indian/Asians (12%), and lowest among Whites (8%) [12].

The prevalence of food insecurity before and during the COVID-19 pandemic is well documented [10,13-18], with some reports indicating that Black African-headed households were more likely to have inadequate access to food compared to Indian-/Asian- or White-headed households [19,20]. However, despite these observations, a critical gap remains in understanding of the underlying socioeconomic factors that contribute to racial disparities in food insecurity and the effects of multiple marginalization on food insecurity among South African adults. To date, no comprehensive analysis has been conducted to quantify the extent to which measurable socioeconomic variables, such as education and employment status, intersect with race to contribute to disparities in food insecurity. While existing studies have highlighted the general patterns of food insecurity across racial groups, they often lack a detailed exploration of how these socio-economic factors interact to create and sustain these inequities. Given the history of apartheid in South Africa, characterised by systemic racial discrimination and limited educational and employment opportunities for those classified as Black Africans [21], and the significant role education plays in socioeconomic outcomes, this study seeks to explore how multiple disadvantages, particularly those related to race, employment and education level, intersect to impact food insecurity in South Africa.

In particular, this study aims to address the following objectives: (i) to examine the socioeconomic inequality in self-reported food insecurity across different racial groups, considering variables such as education, area of residence, and employment status; (ii) to conduct a decomposition analysis to assess contributing measurable socio-economic factors to racial disparities in food insecurity among South African adults. By fulfilling these objectives, this study seeks to contribute to a deeper understanding of the complex interplay between socioeconomic factors and racial disparities in food insecurity during the COVID-19 pandemic. The findings are anticipated to support the development of more effective strategies to mitigate food insecurity and address the socio-economic inequalities that persist within South Africa, particularly in the context of pandemics.

## Methods

**Study design:** this study utilized a cross-sectional design, drawing on data from the 2021 South African Social Attitudes Survey (SASAS). SASAS is a nationally representative survey conducted by the Human Sciences Research Council between October 2021 and January 2022 [22]. SASAS employs a rigorous probability sampling method to ensure a diverse and representative sample across provinces, residence types (rural/urban), and racial groups. The survey follows a multistage probability sampling approach, as previously described and published [22].

**Study setting and population:** the study included South African adults aged 16 years or older from 500 geographically distributed census enumeration areas across all nine provinces.

### Variables

***Household food security*:** household food insecurity was assessed using a previously validated one-item measure [23]. Although this measure was used prior to the COVID-19 pandemic [24,25], it became an acceptable, cost-effective approach for conducting rapid assessments of food insecurity, especially during the COVID-19 pandemic [26,27]. Participants were asked the question, “in the past 12 months, have you ever run out of food and been unable to purchase more” Those who responded “yes” were categorized as food insecure [19].

### Sociodemographic factors

#### Sociodemographic variables

***Self-identified race*:** participants self-identified their race group by selecting one of the following categories: Black African, Coloured, Indian/Asian, and White. These categories were used for analysis without modification.

***Age*:** age was self-reported and categorized into the following groups: 16-24, 25-34, 35-44, 45-54, 55-64, and 65 years or older.

***Gender*:** gender was assessed using the question, “What is your sex?” with response options “male” and “female”.

***Education*:** educational attainment was assessed by asking, “What is your highest level of education?” Responses were grouped into three categories: (1) less than high school (no formal education, less than primary school, or some high school); (2) completed high school (grade 12 or matric); (3) higher than high school (any education beyond grade 12).

***Employment*:** employment status was self-reported and categorized into three groups: employed, unemployed, and not in the workforce (e.g., retired, homemakers, students, or those unable to work due to disability).

***Marital status*:** marital status was grouped into four categories: married, widowed, never married, and separated/divorced.

***Social grants*:** the receipt of social grants was assessed by asking participants whether they received any of the following grants: SRD grant, child grant, disability grant, old age grant, care dependency grant, foster care grant, or grant-in-aid. For analysis purposes, responses were categorized into four groups: SRD grant, child grant, other grants (including disability grant, old age grant, care dependency grant, foster care grant, and grant-in-aid), and no grants.

**Data collection tool:** data were collected through a structured questionnaire administered in face-to-face interviews by trained interviewers. The questionnaire was designed to capture self-reported food insecurity measures from individuals across South Africa in their official languages of preference.

**Sample size:** the initial sample size targeted was 3,500 adults aged 16 years or older. After data collection, 2,837 individuals participated in the survey (81.1% response rate). Respondents with missing data on key variables were excluded, resulting in a final analytical sample of n = 2,488.

**Statistical analysis:** weights were applied to correct for sampling and response biases, ensuring the sample remained representative across demographic subgroups. A Poisson regression model was used to examine the associations between food insecurity and socio-economic factors, adjusting for potential confounders such as race, employment, and education. The model was also used to identify the significant socio-economic factors associated with the prevalence of food insecurity, including gender, age, marital status, area of residence, and receipt of social relief distress grant as potential covariates. Prevalence ratios (PRs) were estimated to quantify these associations. We further examined the interaction between racial groups (other racial groups vs. Black Africans) and employment status on food insecurity in South Africa. Blinder-Oaxaca decomposition analysis of food insecurity gaps between intersectional groups defined by race and socio-economic status.

Building upon the Poisson regression, we employed the Blinder-Oaxaca decomposition model, traditionally used to analyse disparities between groups. This analysis aimed to quantify the extent to which differences in the significant socioeconomic factors identified through Poisson regression contributed to the observed gap in reported household food insecurity between racial groups of interest. This method is particularly well-suited to our study as it allows us to decompose the food insecurity gap between racial groups into explained and unexplained components, offering insights into structural inequalities related to multiple marginalisation [28]. All statistical analyses were performed using Stata version 17 (StataCorp LLC, College Station, TX, USA) to ensure robust and reliable results. Statistical significance was set at p < 0.05.

**Ethical consideration:** this study was performed in line with the principles of the Declaration of Helsinki. Approval was granted by the Ethics Committee of the University of Pretoria (Date: 24 July 2024/No. 451/2023). Informed consent was obtained from all individual participants included in the study. No personally identifiable information was collected, and confidentiality was ensured.

## Results

**Sociodemographic analysis:** a total of 2,837 individuals participated in the 2021 South African Social Attitudes Survey (SASAS) (Table 1). Consistent with the national demographic profile, the majority of participants identified as Black Africans (79.0%), followed by those who self-identified as Coloured individuals (9.0%), Indian/Asians (2.9%), and White participants (9.1%). Other demographic characteristics of the study population are depicted in Table 1.

**Table 1:** sociodemographic characteristics of South African adults aged 16 years or older, South African Social Attitudes Survey 2021 (N = 2,837)

Characteristics	n	Weighted (%)
Total	2836	100
**Race**		
Black Africans	1,738	79.0
Coloured	463	9.0
Indian/Asians	326	2.9
White	310	9.1
**Age**		
16-24 years	334	20.4
25-34 years	628	26.3
35-44 years	647	20.8
45-54 years	460	13.8
55-64 years	403	9.8
65+ years	365	8.9
**Gender**		
Male	1,283	48.1
Female	1,553	51.9
**Education**		
^a^< High school	1227	43.7
Completed high school	1,053	37.8
>High school	499	18.5
**Marital status**		
Married	836	24.5
Separated/divorced	164	4.5
Widowed	341	8.4
Never married	1,353	62.6
**Area of residence**		
Urban	2,279	75.5
Rural	558	24.5
**Employment status**		
Employed	985	33.3
Unemployed	796	33
^b^Not in the workforce	951	33.7
**Social grants**		
Social relief of distress grant	299	11.53
Child grant	583	22.47
No grant	1,108	42.71
^c^Other grants	604	23.28

Note: all percentages (%) are weighted, and all raw counts (n) are unweighted; ^a^: includes those with no school, less than primary school, and those with some high school education; ^b^: includes those retired, homemakers, or very disabled and unable to work, or attending school; ^c^: includes disability grant, old age grant, care dependency grant, foster care grant, and grant in aid

### Descriptive analysis

**Food insecurity among South African adults aged 16 years or older in 2021:** Table 2 presents the bivariate distribution of sociodemographic characteristics and the weighted prevalence of food insecurity among South African adults aged 16 years or older in 2021. Overall, the weighted prevalence of food insecurity was 39.5% (95% CI: 36.02-43.13) in SASAS 2021, based on 944 respondents who reported food insecurity.

**Table 2:** bivariate analysis of sociodemographic characteristics and weighted prevalence of food insecurity among South African adults aged 16 years or older, South African Social Attitudes Survey 2021 (N = 944)

	Food insecurity distribution		
Characteristics	n	Weighted % (95% CI)	^a^p-value
Total	944	39.52 (36.02 - 43.13)	
**Race**			<0.001
Black Africans	671	43.61 (39.62 - 47.69)	
Coloured	149	32.56 (25.18 - 40.92)	
Indian/Asians	71	16.13 (10.35 - 24.27)	
White	53	18.45 (12.80 - 25.87)	
**Age**			0.287
16-24 years	119	43.40 (33.94 - 53.36)	
25-34 years	202	35.78 (30.24 - 41.74)	
35-44 years	214	35.68 (29.36 - 42.53)	
45-54 years	154	39.71 (31.86 - 48.12)	
55-64 years	137	46.51 (38.32 - 54.89)	
65+ years	118	42.21 (34.08 - 50.79)	
**Gender**			0.355
Male	407	37.84 (32.64 - 43.34)	
Female	537	41.08 (36.69 - 45.63)	
**Education**			<0.001
^b^	522	52.0 (47.21 - 56.84)	
Completed high school	319	33.88 (29.40 - 38.66)	
Higher than high school education	92	21.96 (15.45 - 30.23)	
**Marital status**			0.046
Married	233	34.15 (28.94 - 39.77)	
Separated/divorced	64	53.13 (41.40 - 64.52)	
Widowed	117	43.14 (33.73 - 53.07)	
Never married	483	40.01 (35.18 - 45.04)	
**Area of residence**			0.009
Urban	717	36.82 (32.96 - 40.86)	
Rural	227	47.84 (40.63 - 55.14)	
**Employment status**			<0.001
Employed	242	28.75 (24.09 - 33.91)	
Unemployed	347	45.06 (40.05 - 50.17)	
^c^Not in the workforce	328	46.09 (39.85 - 52.45)	
**Social grants**			0.0820
Social distress grant	129	45.00 (36.02 - 54.32)	
Child grant	234	42.99 (37.09 - 49.09)	
No grant	301	33.70 (27.56 40.46)	
Other grants	207	41.13 (34.05 - 48.60)	

Note: all percentages (%) are weighted, and all raw counts (n) are unweighted; food insecurity was defined as participants who responded “yes” to the question: “in the past 12 months, was there any time you have run out of food and not been able to purchase more?”; ^a^: all tests were two-tailed and deemed significant at p^b^ includes those with no school, less than primary school, and those with some high school education; ^c^: includes those retired, homemakers, or very disabled and unable to work, or attending school

Food insecurity varied significantly across racial groups (p<0.001), with the highest prevalence among Black African participants (43.6%, 95% CI: 39.62-47.69), followed by those who self-identified as Coloured (32.6%, 95% CI: 25.18-40.92). Lower prevalence was observed among White (18.5%, 95% CI: 12.80-25.87) and Indian/Asian participants (16.1%, 95% CI: 10.35-24.27).

Significant differences were also observed across education levels (p<0.001). Participants with less than high school education reported the highest prevalence of food insecurity (52.0%, 95% CI: 47.21 - 56.84) while those who attained higher than high school education reported the lowest food insecurity (22.0%, 95% CI: 15.45 - 30.23). Marital status also showed variations in food insecurity (p=0.046). The highest prevalence was observed among participants who were separated or divorced (53.1%, 95% CI: 41.40-64.52), followed by those who were widowed (43.1%, 95% CI: 33.73-53.07). Lower prevalence was observed among married individuals (34.2%, 95% CI: 28.94-39.77).

Differences were also observed by area of residence (p=0.009), with rural residents reporting higher food insecurity (47.8%, 95% CI: 40.63-55.14) compared with those living in urban areas (36.8%, 95% CI: 32.96-40.86). Food insecurity also differed by employment status (p<0.001). Unemployed participants (45.1%, 95% CI: 40.05 - 50.17) and those who were not in the workforce (46.1%, 95% CI: 39.85 - 52.45) reported higher levels of food insecurity compared to those who were employed (28.7%, 95% CI: 24.09 - 33.91).

Although differences were observed across social grant categories, the association between social grant receipt and food insecurity was not statistically significant (p=0.082). Regarding the SRD grant, 45.0% (95% CI: 36.02 - 54.32) of participants receiving the SRD grant reported food insecurity. Among those receiving the child grant, 42.9% (95% CI: 37.09 - 49.09) reported food insecurity. Participants who did not receive any grant reported a lower prevalence of food insecurity at 33.7% (95% CI: 27.56 - 40.46). The prevalence of food insecurity among those receiving other types of grants was 41.1% (95% CI: 34.05 - 48.60). The relationship between social grants and food insecurity was not statistically significant (p=0.082).

**Prevalence of food insecurity by sociodemographic and economic factors:** compared to the White population, the prevalence of food insecurity was 88.0% higher among Black Africans (aPR = 1.88; 95% CI: 1.32-2.69; p = 0.001) and 51.0% higher among Coloured individuals (aPR = 1.51; 95% CI: 1.00-2.26; p = 0.047) (Table 3). Individuals with less than high school education had a 90% higher prevalence of food insecurity (aPR = 1.90; 95% CI: 1.34-2.68; p < 0.001) compared to those with higher education. Food insecurity was 35% higher among those not in the workforce (aPR = 1.35; 95% CI: 1.08-1.69; p = 0.008) and 37.0% higher among unemployed individuals (aPR = 1.37; 95% CI: 1.04-1.56; p = 0.018) compared to the employed. Separated or divorced individuals had a 55.0% higher prevalence of food insecurity (aPR = 1.55; 95% CI: 1.20-2.01; p = 0.001) compared to married individuals. Adjustments for age, gender, area of residence, and receipt of grants did not yield statistically significant associations with food insecurity.

**Table 3:** Poisson regression analysis of factors associated with food insecurity among South African adults aged 16 years or older, South African Social Attitudes Survey 2021 (N = 2,487)

Characteristics			
Race	Prevalence ratios	95% CI	p-value^a^
White (ref)	1		
Black Africans	1.88	1.32 - 2.69	0.001
Coloured	1.51	1.00 - 2.26	0.047
Indian/Asian	0.78	0.46 - 1.33	0.359
**Education**			
>High school (ref)	1		
Completed high school	1.31	0.92 - 1.86	0.131
^b^	1.90	1.34 - 2.68	
**Employment status**			
Employed (ref)	1		
^c^Not in the workforce	1.35	1.08 - 1.69	0.008
Unemployed	1.37	1.04 - 1.56	0.018
**Marital status**			
Married (ref)	1		
Separated/divorced	1.55	1.2 - 2.01	0.001
Widowed	0.98	0.76 - 1.25	0.853
Never married	1.04	0.84 - 1.28	0.726

Food insecurity was defined as participants who responded “yes” to the question: “in the past 12 months, was there any time you have run out of food and not been able to purchase more?”; ^a^: all tests were deemed significant at p^b^: includes those with no schooling, primary school education, and those with some high school education; ^c^: includes those retired, homemakers, or very disabled and unable to work, or attending school

**Blinder-Oaxaca decomposition analysis:** the Blinder-Oaxaca decomposition (Table 4) revealed significant socio-economic contributions to explaining racial disparities in self-reported food insecurity. The overall gap in food insecurity between Black Africans and other racial groups was statistically significant (percentage difference = -12.1%, p < 0.001). Further decomposition revealed that 56.2% of the gap in food insecurity between Black Africans and other races was explained by the two socio-economic factors measured, which include education and employment. Employment alone accounted for about 33% of the difference (56% of the explained contributors) in food insecurity between Black Africans and other races, while education accounted for 17.7% of the gap (30% of the explained contributors). Irrespective of what was accounted for by the racial differences in distribution of these two factors, education and employment remained unexplained (i.e., discriminatory effect) and the largest significant contributor to racial differences in food insecurity, with higher education and employment providing greater advantages to other racial groups than Black Africans (Table 4).

**Table 4:** Blinder-Oaxaca decomposition of food insecurity across intersectional groups defined by self-identified race and socioeconomic status among South African adults aged 16 years or older, South African Social Attitudes Survey 2021 (N = 1633)

Explanatory variables	Coefficient	Proportion contribution (%)	p-value^c^
**Overall model estimates**			
Other race groups (1)	0.258	-	<0.001
Black Africans (2)	0.406	-	<0.001
Difference	-0.148	-	<0.001
Explained	-0.028	18.9	<0.001
Unexplained	-0.120	81.1	<0.001
**Factor contributions**			
**Explained**			
Employment	-0.013	8.8	p<0.000
Education	-0.009	6.1	0.001
**Unexplained**			
Employment	0.037	30.8	0.014

Food insecurity was defined as those who indicated either themselves or household members skipping a meal because there is not enough money for food “once a month” “less than once a month” “once a month” “several times a month” “once a week” “several times a week”; ^c^: all tests were deemed significant at p<0.05

**Food insecurity by race, stratified by employment:** food insecurity varied by race and employment status. Among employed individuals, Black Africans experienced a higher prevalence of food insecurity (32.7%) compared to individuals from other race groups (19.8%). This disparity was even more pronounced among the unemployed, with food insecurity affecting nearly half (48.9%) of unemployed Black Africans, compared to 28.5% of unemployed individuals from other race groups (Figure 1).

**Figure 1:**
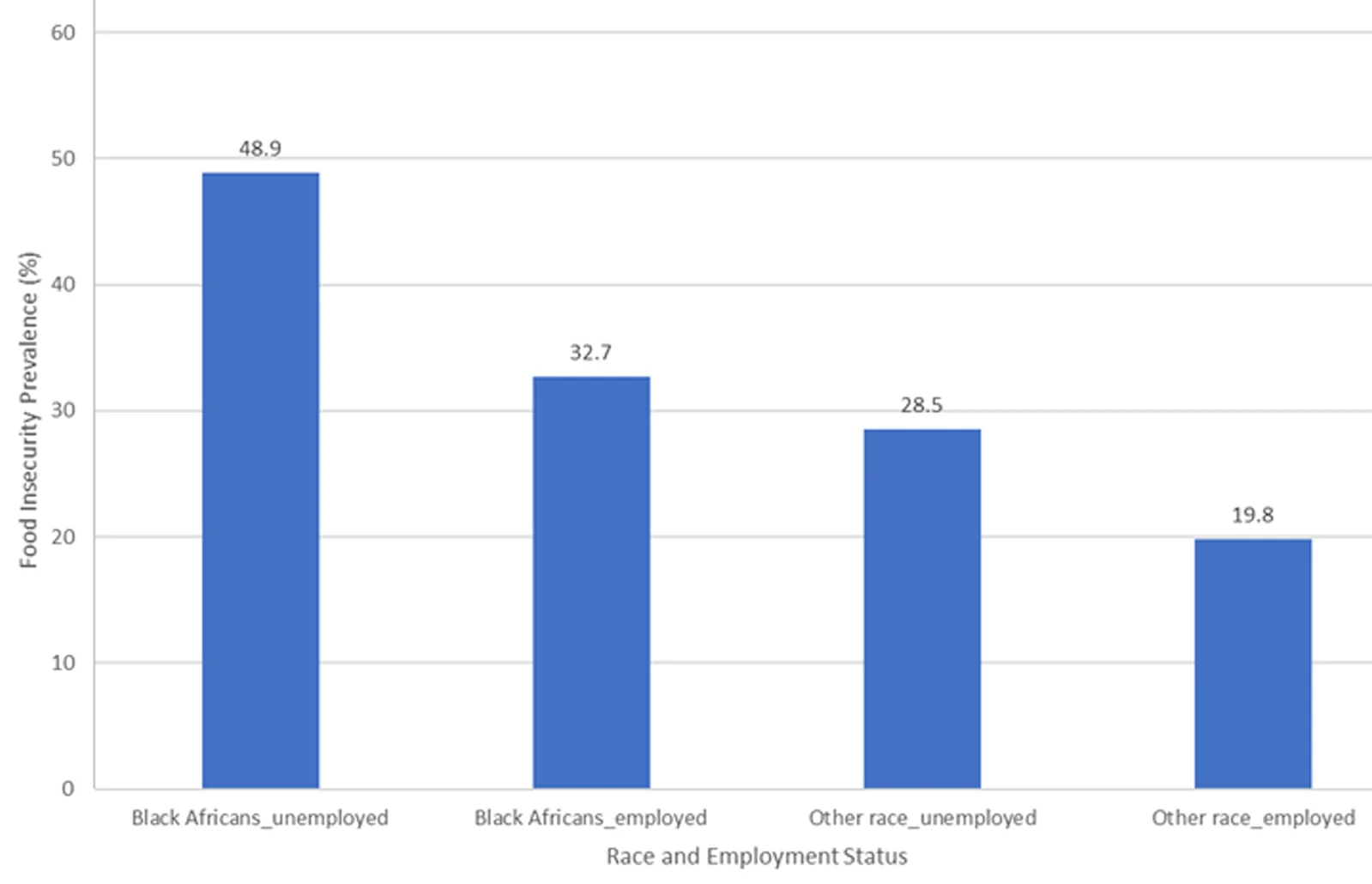
prevalence of food insecurity when comparing groups defined by the intersection between different racial groups and employment status

## Discussion

The findings of this study highlight the persistent racial and socio-economic disparities in food insecurity across South Africa, with Black African communities reporting the highest prevalence of food insecurity. In particular, Black Africans were almost twice as likely to experience food insecurity compared to White individuals. These findings are consistent with previous research, highlighting the disproportionate impact of food insecurity on Black Africans during the COVID-19 pandemic [10,14,16,29-31].

The decomposition analysis sheds light on the observed differences in food insecurity between Black Africans and other racial groups. This statistical method separates the differences into explained components (attributable to factors like education and employment) and unexplained components (which may suggest discriminatory impacts on race). Our findings show that almost 20% of the observed differences can be attributed to unequal distributions in educational attainment and employment levels between the racial groups. This is consistent with existing literature, which highlights that, on average, Black Africans have lower levels of educational attainment and are more likely to face unemployment compared to other racial groups, particularly compared to their White counterparts [32,33]. Additionally, job losses and economic hardships were more pronounced among Black Africans with education below high school during the COVID-19 pandemic [34], intensifying their structural vulnerability to poverty and food insecurity. The racial disparity in food insecurity persisted despite the provision of the SRD grant, which was predominantly received by individuals who were unemployed, students, employed in casual work, or those with a high school education or less [7].

Consistent with this observation, a substantial portion of employment-related disparities remained unexplained in the decomposition model, suggesting the presence of a discriminatory effect of employment on race. Consistent with previous reports, this implies that employment does not provide the same level of protection against food insecurity across all racial groups [35,36]. The stratified analysis confirmed these findings, revealing that even at similar employment levels, Black Africans faced disproportionately higher levels of food insecurity compared to individuals from other racial groups. These results highlight the intersection of race and employment status in shaping food insecurity outcomes.

These findings further underscore that, beyond the explained contributing factors in our decomposition analysis, additional social and historical contexts may contribute to the observed disparities in food insecurity. Structural barriers, rooted in apartheid-era policies and ongoing discriminatory practices, such as systemic racism [35,37], have sustained income inequalities for Black Africans, in comparison to other race groups, most notably their white counterparts [35]. South Africa remains one of the most unequal countries globally, with employed Black Africans earning the lowest wages relative to other race groups [35,36]. For instance, the 2022/23 income and expenditure survey shows that the average household income of White-headed households was nearly five times higher than that of Black African-headed households [37]. Furthermore, a study by Feder and Yu [38] found that low-wage employment and working poverty were predominantly experienced by Black South Africans. Consequently, although Black African participants in this study reported being employed, their earnings may have been insufficient to protect them from food insecurity.

Beyond income disparities, apartheid-era exclusionary policies systematically denied Black Africans access to long-term assets such as land ownership and economic opportunities [21,39-41], creating a persistent racial wealth gap [42]. In contrast, individuals from other racial groups may benefit from generational wealth, investments, or assets that could serve as a financial buffer against food insecurity, even during periods of unemployment [43]. For instance, government agricultural subsidies have mainly favoured large-scale, predominantly White commercial farmers, limiting opportunities for Black-owned agricultural enterprises, and restricting local food production in Black communities [44]. This lack of support has compounded over generations, reducing Black Africans´ economic resilience and increasing reliance on expensive, commercially sourced food. Consequently, Black African families continue to face systemic barriers that make escaping poverty and achieving food security increasingly difficult [45,46]. While some who self-identify as being from other racial groups, such as Indians, have faced economic barriers, the impact has been far more severe for Black Africans [41].

Other factors not accounted for in our analysis include disruptions of informal markets during the COVID-19 pandemic, which primarily serve low-income communities, particularly Black African communities. These disruptions limited access to affordable and fresh produce [47]. Transportation constraints further hindered rural and informal urban residents´ ability to access food supplies [11,37], while rising food prices exacerbated food insecurity for these populations [9,48].

Additionally, geographical policies have historically marginalized rural areas and townships, where Black populations predominantly reside, by depriving them of basic infrastructure like grocery stores and transportation. This systemic neglect has contributed to the creation of food deserts, compounding the issue of food insecurity in these communities [49].

Another important consideration is the informal family support structures within Black African communities, which can lead to increased financial burdens through the practice of 'Black tax.' This practice involves one or more employed individuals within a family, shouldering the responsibility of supporting extended family members [39]. This financial strain is further intensified by student loan debt, such as obligations from government programs like the National Student Financial Aid Scheme (NSFAS), which primarily supports those who would otherwise be unable to afford higher education [45,50]. In 2019, for example, 93.9% of NSFAS-funded students were Black Africans [50]. Although tertiary education can improve earning potential, student loan repayments create significant financial pressure, particularly among employed Black Africans. These additional financial demands on already limited resources may further compromise individual and household food security. While these factors were not included in our analysis, they are critical considerations for future research to better understand the broader structural determinants of food insecurity.

Our findings highlight that addressing food insecurity disparities requires more than improving education and employment for Black Africans. While these factors are important, they alone cannot overcome the other structural barriers perpetuated by systemic racism and historical disenfranchisement. Policymakers must implement structural reforms to address the legacy of apartheid, enhance access to quality education and employment (salary parity), and rectify inequalities in land ownership and agricultural policies. Without these broader systemic changes, efforts to reduce food insecurity will be insufficient, and racial disparities will likely persist. To effectively address food insecurity among multiply marginalized groups, it is crucial to consider both socio-economic and systemic factors, and further research is needed to develop tailored interventions for future economic shocks.

**Limitations and future directions:** this study's findings may lack generalizability beyond the specific context of the COVID-19 pandemic, as the data were collected during this unique period. The economic and social disruptions caused by the pandemic may have disproportionately influenced food insecurity rates, particularly among Black Africans. Therefore, these results may not fully reflect pre-pandemic trends or post-pandemic recovery. Furthermore, the cross-sectional design restricts our ability to establish causal relationships between race, education, and food insecurity. The associations observed in this study should be interpreted cautiously, as temporal sequencing cannot be determined. However, since food insecurity was measured after educational attainment and employment, the association reported here is less likely to be affected by the lack of information on the sequence of events. Nonetheless, the use of a single-item measure for food insecurity as well as self-reported data on food insecurity may reduce validity and introduce reporting biases. Lastly, the Poisson models did not capture all possible factors contributing to food insecurity. For instance, additional factors like mental health, access to transportation, and food deserts could influence the observed disparities, but were not collected in the original survey. This limitation may affect the accuracy of the unexplained portion in the decomposition analysis. Future research should consider employing multilevel and longitudinal designs, which can track changes in food insecurity over time and provide clearer insights into cause and effect that are contextual to where people live. Additionally, using multiple-item measures of food insecurity and including related variables such as transportation access and geographic disparities (e.g., food deserts), including estimates of food obtained from subsistence farming, could provide a more nuanced understanding of the complex factors contributing to racial disparities in food insecurity in South Africa.

## Conclusion

Our study reveals significant racial disparities in food insecurity, particularly among Black Africans, who face higher rates of food insecurity than other racial groups despite employment status. This finding highlights that employment status does not provide equal protection against food insecurity across racial groups, suggesting the presence of systemic barriers and discriminatory effects within the labour and economic system. Addressing these disparities requires targeted policy interventions that go beyond promoting access to education and employment to dismantle the structural inequities perpetuating these outcomes. Future research should further explore these barriers to inform tailored strategies that ensure equitable food security for all racial groups.

### What is known about this topic

- The COVID-19 pandemic exacerbated food insecurity globally, with socio-economic gaps widening, particularly in South Africa;
- Racial disparities in food insecurity have been documented, with Black Africans being disproportionately affected;
- Socioeconomic factors such as employment status are critical in understanding food insecurity, but the intersectionality of race and socio-economic status requires further exploration.

### What this study adds

- The study highlights that Black Africans have significantly higher food insecurity compared to White individuals, even after adjusting for socio-economic factors;
- Decomposition analysis reveals that employment status accounts for a substantial portion of the racial food insecurity disparity, but unexplained (discriminatory) factors remain;
- This study emphasizes the complex interplay of race and socioeconomic status in food insecurity, highlighting the need for targeted policy interventions addressing multiple forms of marginalization.
